# Atlanta Medical Society

**Published:** 1866-04

**Authors:** S. H. Stout, H. S. Wilson

**Affiliations:** President; Atlanta; Secretary; Atlanta


*Atlanta Medical Society.*—S. H. Stout, M. D., President—H. S. Wilson, M. D., Secretary.

Atlanta, February 13, 1866.

On the call for reports of cases, Dr. Word mentioned the fact that he had in preparation, the report of a case of Placenta Prævia, which he proposed to present in the form of a written report, at some future meeting.

*Dr. J. G. Westmoreland*, without making a formal report of any particular case, asked permission to call the attention of the Society to the subject of Variolous influence upon the fœtus in utero, and elicit the opinion of members in regard to the period of the disease in the mother, at which the fœtus becomes impressed; and whether or not, the mother and fœtus receive the contagion at the same time.

*Dr. John Boring*, in response, reported a case in which a female had been vaccinated four weeks before delivery, (whether successfully or not, he was not informed). Three weeks after the alleged vaccination, and eight days before delivery, the eruption of variola appeared. Four days after the birth of the child, and twelve days after the eruption appeared upon the mother, it also exhibited the eruption of small-pox. This case evidently affords proof in support of the opinion that the disease is propagated in the fœtus at the period of its full development in the mother; the time being about fifteen days of incubation for the child, counting from the febrile stage before eruption in the mother, to the eruption in her offspring. This is about the average period of incubation, and the fœtus evidently was impressed with the disease about that time. The length of time from the reception of the virus by the mother to the development of the disease in the child, allowing fifteen days of incubation in the mother, would be about thirty days, entirely too long for the period of incubation.

*Dr. W. F. Westmoreland*, in connection with this subject, reported a case of small-pox in a woman, at about the seventh month of utero gestation. Three weeks after, when desquamation was complete, premature delivery occurred. The child, when born, had the eruption of variola, apparently of about eight days duration. These facts would fix the time of contracting the disease by the child, at about the febrile or eruptive stage in the mother, allowing the usual period of incubation.

*Dr. Alexander* desired, while the subject of small-pox was being considered by the Society, to hear the views of members in regard to small-pox being communicated before the eruption appears. He considered the question one of importance to the profession, inasmuch as the community feel a deep interest in getting at the truth on this subject. He had sufficient evidence to form an opinion against the probability of communicating the disease before the eruption appears.

*Dr. Hillyer* offered some theoretical views and arguments in support of Dr. Alexander's opinion.

*Dr. W. F. Westmoreland*, Dr. Conally, and other members, believed that at any time during the febrile stage, before eruption, the disease could be communicated to others. Cases were cited by the advocates of each opinion, and on motion the opinion of each member was requested, when only ten had come to any satisfactory conclusion on the subject. Six believed it communicable before the eruption, and four, that it was not.

---

MARCH 6, 1866.

The Report of cases being in order,

*Dr. Alexander* reported a case of labor, in which chloroform was used, causing, as it would seem from this and others previously noticed, the suspension of lactation. He desired other members of the Society to give their experience in the use of this valuable remedy in regard to its effect in this way. No member had noticed this result ; but in alluding to the effects of chloroform in obstetrical practice, several members alluded to the antihemorrhagic action, not only upon the uterus, but other organs. For this purpose it had been the custom of members to administer the remedy by the stomach, in half drachm to drachm doses, suspended in acacia mucilage. The subject taking a wider range, the general effects and uses of chloroform were discussed.

Other reports being called for,

*Dr. R. C. Word* made the following

### REPORT OF A CASE OF PLACENTA PRÆVIA.

Visited Mrs. H. first on the 16th March, 1861. Pregnant with her third child. Symptoms, pain in the back and some pain in the abdomen, which had existed for several hours with considerable flooding, though not sufficient to weaken, materially, the force of the pulse. She appeared somewhat flushed and excited, yet without undue heat or fever. Two weeks previously she had had similar symptoms. She was at the eighth month, and having miscarried in the previous pregnancy, we thought she was again threatened with a like result—an impression which was strengthened rather than removed by a vaginal examination, which revealed a soft and spongy condition of the cervix uteri exceedingly sensitive and tender to the touch, leading to the inference that the supposed threatened miscarriage was due to a diseased state of the os. The finger penetrated the neck an half inch or more, but did not reach the membranes or fœtus. To pressure upon the abdomen the womb felt unusually soft and flabby, and on applying the ear, could distinctly hear the fœtal heart on the right side of the mother. ℞—Patient to keep cool and quiet, and take one-third of a grain of morphine every three hours, until pain and hemorrhage ceased.

On the 19th, three days subsequent to last visit, again saw her. The flooding had returned to-day and the liquor amnii was discharging, but no pain indicative of labor. On examination found the mouth of the womb somewhat dilated, and the entire vagina and neck very tender. The placenta was detected directly over the os uteri. ℞—Patient to keep cool and quiet—cold applications to vulva; acid drinks and small doses tinct. opii oft repeated. To be sent for if at any time the flooding becomes alarming.

On the 20th, about thirty hours after, again saw her. The hemorrhage had recurred at intervals, and for a few hours past had largely increased, attended with uterine pains and slight bearing down tendency. The mouth of the womb was soft and dilatable, from which was pending a fold of the umbilical cord plainly pulsating. Decided to turn and deliver. Patient on her back—hips on the edge of the bed, and each foot in a chair. Partial anæsthesia was induced with a mixture of one part ether and two of chloroform. The right hand well lubricated with lard, and dipped in warm water, was slowly and cautiously introduced. On reaching the placenta, found it more convenient to penetrate than to detach and pass round it, though there was considerable difficulty in rupturing the membranous envelop. The head was found above the brim with occiput toward the right acsetabulum. First grasped a shoulder—supposing it to be a knee—then with some delay and difficulty found the left foot and brought it down—manipulating externally with left hand to aid the turning. The left foot was entirely without the vulva before the right could be found. There was now some pain. We gave a dose of ergot, and trusted for a time to the natural efforts of the womb. The pains became stronger. No hemorrhage. During the pains we assisted by tractoin upon the fœtus, and the case progressed favorably until the head reached the rent in the placenta, when the pains suddenly ceased, followed immediately by great restlessness and distress on the part of the patient, with alarming evidences of exhaustion and sinking, the pulse being scarcely perceptible. The patient was ordered to be placed full length in bed, and hot toddy and ergot was freely administered for twenty or thirty minutes, but no reaction following, the fœtus was then drawn forcibly and somewhat violently through the rent in the placenta, passing it with a sort of jerking sensation, and was promptly delivered, and the placenta also immediately removed. The patient began to rally in a few minutes, though there was tendency to flooding for more than an hour, which was treated with acid drinks and black drop internally, and cold applications to the abdomen, and warmth to the extremities.

It is scarcely necessary to mention that the child was dead. The time occupied in the delivery was one and a half hours, and the quantity of the anæsthetic inhaled, about two ounces.

This patient had a good recovery, being able to sit up in a few days.

The points of comment in this case are the following:

1st. The exceeding sensitiveness of the os tincæ and the vagina.

2d. The difficulty of the head passing the rent in the placenta, and the great and dangerous constitutional disturbance caused by it.

3d. The probability of a shoulder presentation in this case, had the placenta been removed, and the labor trusted to the natural powers as recommended by some authors.

With regard to the exceeding tenderness of the os uteri and the vagina, we are not aware that it is mentioned by any author as a symptom peculiar to placenta prævia; yet in this case it was so great as to furnish an almost insuperable obstacle to a careful and proper examination of the parts. To such a degree indeed did it exist, that it misled me in the first examination, inducing the belief that there was cancerous or other diseased state of the os, from which the hemorrhage proceeded, and which was the cause of a supposed threatened miscarriage. If this condition exists in every case, it should be recorded as an important diagnostic symptom.

The obstacle furnished by the placenta, to the passage of the head, is another point overlooked by the authorities, and which is well worthy the attention of the profession, as it may furnish an argument against the mode of delivery by turning, and in favor of the plan advised by Prof. Simpson and others, of first delivering the placenta and then trusting the labor to the natural powers; for though the precaution of making a large and free rent in the placenta might, to some extent, lessen the obstacle, yet it is manifest that so large a body as the placenta, located in the narrow area of the neck and brim, must always furnish a serious obstruction to the delivery of the body and head of the fœtus. Nor would the plan of detaching and passing around the placenta to reach and bring down the feet, better the case, but rather increase the difficulty; for it would be easier by traction upon the fœtus, to distend and enlarge an opening already made, than to overcome or tear away the entire lateral half as would seem to be necessary in the latter operation.

The great and alarming constitutional disturbance occasioned by the obstruction to the advance of the child in this case, may happen in any case of obstructed labor; yet, as the books make no mention of it in placenta prævia, it is likely to alarm and mislead the young practitioner. Under the head of "Symptoms of Powerless Labor," Dr. Dewees notices, at some length, this peculiar constitutional disturbance as indicative of obstructed labor. We have often observed this condition. It is characterized by an increased frequency of pulse, nausea, vomiting, hot skin, dry tongue, restlessness, hot vagina, an expression of great anxiety and alarm, with a sense of sinking. Such were the symptoms in this case. Our first impression was, that the patient was simply exhausted, or had taken too much chloroform, but finding that rest and stimulants did not relieve the patient, we immediately suspected that an obstruction had occurred, beyond the powers of the patient to overcome, and by using the necessary force to overcome the obstacle, had the satisfaction of seeing this opinion confirmed by an immediate reaction on the part of the patient, with relief, in a short time, from all the distressing symptoms.

Upon the third point we would remark, that the presence of the placenta in advance of the child, and occupying the space where the head properly belonged, appeared to interfere with the necessary adaptation of the occiput to the axis of the superior strait, and thereby to encourage a shoulder presentation. Such would undoubtedly be the tendency were the contractile efforts of the womb violent. If, then, the plan of first removing the placenta and trusting the case to nature were adopted, in any case, it would be well to remember this fact, and give early attention to the position of the head.

Upon the whole, the impression made upon my mind by this case is, that in every instance, where the placenta is fully over the os uteri, that turning should be resorted to as soon as the parts are in a condition to allow it, and under the relaxing influence of chloroform, this may be done sooner than is usually advised; that the placenta should be first hurriedly detached and removed, and the feet brought quickly down, and the body of the child brought in prompt apposition to the open and bleeding sinuses left by the placenta, in order to arrest the hemorrhage and save the strength of the patient. If, at this stage of the proceeding, there should, from any cause, be reason to believe that the child is dead beyond any hope of resuscitation, the case may here be trusted to nature, and conducted as an ordinary foot presentation. But if the delay has not been long, and there is hope for the child, then the delivery should be as prompt as may be consistent with the safety of the mother.

If the detachment and removal of the placenta could in every instance be relied upon as a means of arresting the hemorrhage, then it becomes an important question whether, after so detaching it, the mother should be subjected to the operation of turning, in the hope of saving the child by a more rapid delivery than could be expected from the natural efforts. We think that the life of the mother should not be too greatly jeopardized to save the fœtus; yet, it does not appear from the limited number of cases reported that we can calculate with certainty upon the cessation of hemorrhage on the removal of the placenta. On the contrary, in a considerable proportion of the cases, the flooding was not arrested, and turning, or other interference, was found necessary to hasten the delivery. But under the plan above proposed, the hemorrhage would not only be arrested, but in all cases where the parts were in good condition, the pelvic capacity ample, and the fœtus relatively small, thereby making a rapid delivery practicable, we opine that the chances of life to the child would be greatly enhanced, while the danger to the mother would not be materially increased.

